# Improvement of the Bonding Properties of Mineral Trioxide Aggregate by Elastin-Like Polypeptide Supplementation

**DOI:** 10.1155/2019/3484396

**Published:** 2019-08-19

**Authors:** Hyun-Jung Kim, Donghyun Lee, Seungryong Cho, Ji-Hyun Jang, Sahng Gyoon Kim, Sun-Young Kim

**Affiliations:** ^1^Department of Conservative Dentistry, Kyung Hee University Dental Hospital, Seoul, Republic of Korea; ^2^Department of Nuclear and Quantum Engineering, KAIST, Daejeon, Republic of Korea; ^3^KI Institutes for IT Convergence, Health Science and Technology, and Artificial Intelligence, KAIST, Daejeon, Republic of Korea; ^4^Division of Endodontics, Columbia University College of Dental Medicine, New York, NY, USA; ^5^Department of Conservative Dentistry and Dental Research Institute, School of Dentistry, Seoul National University, Seoul, Republic of Korea

## Abstract

**Introduction:**

Elastin-like polypeptide (ELP) supplementation was previously reported to enhance the physical properties of mineral trioxide aggregate (MTA). The aim of this study was to investigate the effect of ELP supplementation on the bonding properties of MTA to dentin.

**Methods:**

Two types of ELPs were synthesized and mixed with MTA in a 0.3 liquid/powder ratio. The push-out bond strength test and interfacial observation with scanning electron microscopy were performed for ELP-supplemented MTA. The porosity of MTA fillings in the cavity was observed with microcomputed tomography. The stickiness, flow rate, and contact angle were additionally measured for potential increased bonding properties.

**Results:**

ELP supplementation improved the bond strength of MTA to dentin. MTA supplemented by a specific ELP exhibited a less porous structure, higher stickiness, and higher flow rate. ELPs also decreased the contact angle to dentin.

**Conclusions:**

This research data verifies that ELP improves the bonding properties of MTA to a tooth structure. The sticky and highly flowable characteristics of ELP-supplemented MTA may provide intimate contact with dentin and supply a less porous cement structure, which might improve the bonding properties of MTA.

## 1. Introduction

Mineral trioxide aggregate (MTA) has been used to repair discontinuity of tooth structures caused by severe caries, trauma, and iatrogenic accidents due to its favorable properties such as high biocompatibility and good sealing ability [1, 2]. One typical example of discontinuity is a pulp exposure, which is the abrupt appearance of soft tissue inside the tooth due to the breakdown of dentin continuity through mechanical or pathological reasons. Another discontinuity in a tooth structure is root perforation, which is an artificial communication between the root canal system and periradicular tissues of the teeth [3].

Despite the long use of MTA as a good reparative material, its use is limited to only small defects [4, 5]. Large-size pulp exposures and root perforations can be more difficult to repair and seal, potentially allowing continuous bacterial contamination and irritation at the perforation site [6, 7]. Overextensions to periodontal tissue and washouts of MTA may occur easily on large defects due to the slow setting time and low mechanical properties of MTA at the initial stage [8, 9]. To overcome these limitations, some studies reported the use of an internal matrix, such as a calcium sulfate barrier and resorbable collagen for the repair of large defects [3, 10]. However, a successful treatment of large discontinuities of the tooth structure remains a challenge.

An elastin-like polypeptide (ELP) is a popular protein-based biomimetic polymer developed using genetic engineering technology. ELPs consist of repeating amino acid sequences of Val-Pro-Glu-Xaa-Gly, where Xaa is a guest amino acid except Pro [11, 12]. ELPs have elasticity and resilience similar to those of *in vivo* elastin and are so highly biocompatible that they do not cause an immune response [13, 14]. ELPs have been widely used in cancer therapy and drug delivery and as a regenerative tissue scaffold in the medical field [11]. Recently, a previous study showed that ELP supplementation improves the physical properties of MTA [15]. If MTA has a bonding property to a tooth structure in addition to its improved physical properties, enhanced retention on the tooth substrate and reinforced hermetic sealing along the margins could result. Furthermore, the enhanced properties would increase the availability of its use as a reparative material for large defects. Some studies have demonstrated the possibility of bonding properties in ELP-supplemented inorganic cement. Murphy et al. reported the feasibility of using ELPs to function as a polymeric matrix to fix hydroxyapatite crystals [16]. Jang et al. reported that viscous characteristics were observed at the same liquid/powder (L/P) ratio when mixing inorganic cement with an ELP solution [15]. However, whether ELP supplementation in inorganic dental cement exhibits any improvement in bonding to the tooth structure has yet to be concluded.

Therefore, the aim of this study was to investigate the effect of ELP supplementation on improvement in bonding properties of MTA to dentin. We evaluated the bond strength of MTA to dentin and also performed supportive experiments to explain the reasons for the improved bonding properties in ELP-supplemented MTA.

## 2. Materials and Methods

### 2.1. Material Preparation

Two types of ELPs, V125 and V125E8, were prepared using a method from a previous study [15]. Briefly, DNA sequences for V125 and V125E8 were created and transferred to modified pET28b vectors. The ELP plasmids were transformed into BLR (DE3) *E.coli* and purified using inverse transition cycling. A 10% solution of ELP by weight in deionized water (DW) was used as the liquid phase to achieve optimal handling and mechanical properties [12]. The L/P ratio of ELP solution to MTA (ProRoot MTA; Dentsply Tulsa Dental, Tulsa, OK) was 0.3 throughout the following experiments.

### 2.2. Measurement of Push-Out Bond Strength and Evaluation of the Debonded Surface

Extracted caries-free human third molars were selected for this study. The experimental protocol using human teeth was reviewed and approved by the Institutional Review Board (KHU-1808-1). The midcoronal portion of the tooth was horizontally cut into 2 mm thick dentin discs using a water-cooled high-speed saw (IsoMet 5000; Buehler, Lake Bluff, IL) (*n* = 12). A cylindrical cavity 1.5 mm in diameter was prepared in each dentin disc with a depth-cutting bur (Microcopy, Kennesaw, GA). The dentin discs were soaked sequentially in 17% EDTA and 2.5% sodium hypochlorite for 1 minute each and finally washed with phosphate-buffered saline and dried [17]. MTA was mixed with either DW, V125, or V125E8 solution and carefully loaded into the cavity without compaction pressure. The specimens were stored at 37°C with 100% humidity conditions for 48 hours and went through a 1-week maturation period in simulated body fluid (SBF) in a 37°C incubator [18].

The push-out bond strength was measured using a universal testing machine (AGS-X; Shimadzu, Tokyo, Japan) at a crosshead speed of 1.0 mm/min. The maximum load applied to the MTA mixture before dislodgement was divided by the total contact area of the cavity in mm^2^ to obtain a value in MPa.

The discs were then examined under a light stereo-microscope (Sunny, Shanghai, China) at 40x magnification to determine the mode of failure. Each sample was classified into one of two failure modes: adhesive failure and mixed failure; there was no complete cohesive failure. The debonded inner surfaces with an adhesive failure mode were fractured in half and examined under a scanning electron microscope (SEM) (JSM-840; JEOL, Tokyo, Japan) after gold sputter coating.

### 2.3. SEM Evaluation of the Dentin-MTA Mixture Interface

Dentin discs 4 mm in thickness from the third molars were fabricated with a high-speed saw (IsoMet 5000). To evaluate the MTA mixture-dentin interface in a closed cavity, a cylindrical cavity was prepared to a diameter of 2 mm and a depth of 2 mm with one end open in each dentin disc. After careful loading of the MTA mixture without packing pressure, the specimens were stored using the same method as in the push-out bond strength experiment. The specimens were dried, coated with nail varnish on the top surfaces, and then embedded in epoxy resin. The specimens were then cut along the center to expose the internal interface between the MTA and tooth wall using a low-speed diamond saw (Isomet; Buehler) under water irrigation. The exposed internal surfaces were serially polished with SiC papers (#400–4000 grit). The smear particles were removed by soaking in 17% EDTA solution for 1 minute. Samples were dried at ambient temperature for 24 hours. The interfaces were then observed under a SEM (JEOL) after gold sputter coating.

### 2.4. Microcomputed Tomography (micro-CT) Evaluation of Porosity

Specimens for micro-CT analysis were fabricated by the same procedure as for push-out bond strength measurement and stored using the same method (*n* = 9). The specimens were then analyzed using a micro-CT scanner (Skyscan1272; Bruker, Kontich, Belgium) with a 360° angle and a pixel size of 2 *μ*m. To quantitatively assess the empty space in set MTA mixtures including the gap between the MTA mixture and cavity wall, we measured the volume of air gaps and that of the cement structure. Following 3D reconstruction of micro-CT images with the Feldkamp-Davis-Kress algorithm [19], image segmentation was performed to compute the volumes of air gaps and cement. After delineating the cylindrical volume, the air gaps were separated from the cement using Otsu's threshold method, with the threshold selected empirically from the histogram of voxel values [20]. The empty space (*Φ*) was then calculated with the following formula:

(1) $$\begin{array}{c} \phi =\frac{V_{A}}{V_{A}+V_{C}}, \end{array}$$

where *V*_*A*_ and *V*_*C*_ represent the volume of the air gap and the volume of the cement, respectively. The image processing and quantitative evaluation programs were coded in the Matlab (R2017a) platform.

### 2.5. Measurement of Stickiness

Dentin discs 2 mm in thickness were cut from extracted human third molars and serially polished with SiC papers (#400–4000). The dentin discs were then soaked in 17% EDTA solution for 1 minute, dried, and fixed on a glass plate with an adhesive tape (3 M, St. Paul, MN, USA). A Texture Analyzer (TA XT plus; Stable Micro Systems, Surrey, UK) equipped with a 100 g load cell, and a flat stainless steel probe 10 mm in diameter was used for measurements. A 0.3 mL quantity of each experimental mixture was carefully loaded onto the dentin disc (*n* = 3). After 1 minute, the probe was slowly moved downward (1.0 mm/sec) to squeeze the mixture until the final gap between the probe and dentin disc was 0.1 mm. The probe was subsequently pulled back at a crosshead speed of 1.0 mm/sec. The force required for separation was recorded to an accuracy of 0.1 mN.

### 2.6. Measurement of the Flow Rate of the MTA Mixture

The flow rate of the MTA mixture was tested according to the ISO 6876 criteria. A 0.5 mL quantity of mixed paste with a 0.3 L/P ratio was placed on the center of a glass plate with a dimension of 70 × 70 mm^2^. After 3 minutes, a 100 g glass plate with the same dimension was placed on top of the material. After 10 minutes of MTA mixing, the minimum and maximum diameters of the sample were measured with a digital caliper (Mitutoyo Corp., Kanagawa, Japan) with a resolution of 0.01 mm (*n* = 3).

### 2.7. Measurement of the Contact Angle on the Dentin Surface

Dentin discs 2 mm in thickness were cut from extracted human third molars and polished gently with serial SiC papers (#400–4000 grit) under water until a flat surface was obtained and were then soaked in 17% EDTA solution for 1 minute. A drop of each liquid (DW, 10 wt% V125, and V125E8 solution) was deposited on the prepared dentin disc, and the contact angle was measured by an image analyzer equipped with an installed video camera (Phoenix 300; Surface Electro Optics, Suwon, Korea) (*n* = 5). The contact angle of the liquid droplet was calculated by averaging the contact angles of the left and right sides.

### 2.8. Statistical Analysis

The push-out bond strength (N), porosity (%), stickiness (mN), flow rate (mm), and contact angle (°) data were analyzed by the one-way analysis of variance. A Bonferroni test was used for the *post hoc* analysis. The level of significance was set at *α* = 0.05. All statistical analyses were performed with SPSS 22.0 (IBM Corp, Armonk, NY).

## 3. Results

### 3.1. Measurement of Push-Out Bond Strength and Evaluation of the Debonded Surface

**Figure 1:**
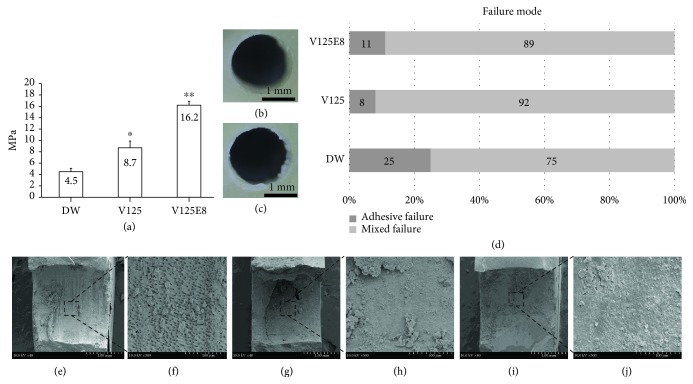
Push-out bond strength, distribution of failure mode, and SEM evaluation of adhesive failure: (a) push-out bond strength; (b, c) microscopic views of adhesive failure and mixed failure, respectively; (d) distribution of failure modes; (e, f) lower and higher magnification views of the adhesive failure of DW; (g, h) lower and higher magnification views of the adhesive failure of V125; (i, j) lower and higher magnification views of the adhesive failure of V125E8. ^∗^*P* < 0.05 compared to the DW group; ^∗∗^*P* < 0.05 compared to both the DW and V125 groups.

The V125E8 group exhibited the highest push-out bond strength, while DW had the lowest (*P* < 0.05) (Figure 1(a)). Two types of failure modes (mixed and adhesive failures) were observed at the debonded surface (Figures 1(b) and 1(c)). Mixed failure was mostly common in all groups (Figure 1(d)).

The representative SEM images of the debonded surfaces among adhesive type failures are presented in Figures 1(e)–1(j). Although all groups commonly exhibited attached MTA pieces on the surface, open dentinal tubules were often observed in the DW group and rarely in the V125 and V125E8 groups.

### 3.2. SEM Evaluation of the Dentin-MTA Mixture Interface

**Figure 2:**
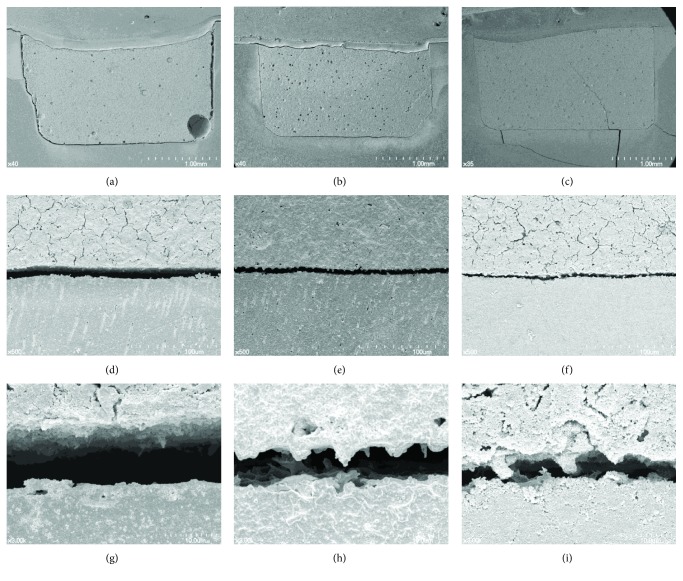
Representative SEM images of the dentin-MTA interface: (a, b, c) lower magnification of the interface of DW, V125, and V125E8, respectively; (d, e, f) higher magnification of the interface of DW, V125, and V125E8, respectively; (g, h, i) highest magnification of the interface of DW, V125, and V125E8, respectively.

The representative SEM image of the dentin-MTA mixture interface is shown in Figure 2. V125E8 exhibited the narrowest interfacial gap (approximately 2-3 *μ*m), while DW demonstrated the widest gap (approximately 10 *μ*m) with V125 in the middle (approximately 5-6 *μ*m). V125 and V125E8 at higher magnification seemed to have more tag-like structures or rugged surfaces on the side of MTA compared to the DW group.

### 3.3. micro-CT Evaluation of Porosity

**Figure 3:**
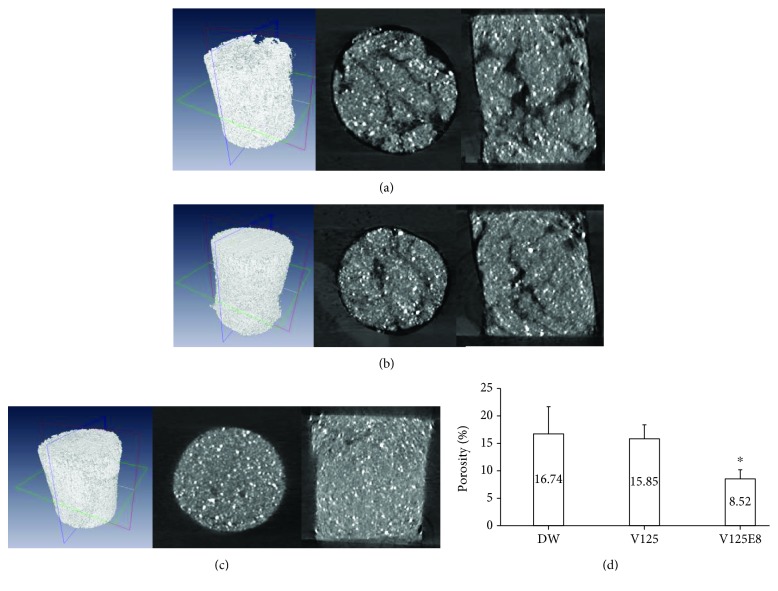
Porosity evaluation through micro-CT. (a, b, c) Serial images of the three-dimensional, cross-sectional, and longitudinal views for DW, V125, and V125E8, respectively. (d) Porosity (%) of the MTA mixtures (*n* = 9). ^∗^*P* < 0.05 compared to the DW group.

3D images of the three experimental groups in the cylinder-like cavity were reconstructed (Figures 3(a)–3(c)). In both longitudinal and cross-section views, DW and V125 exhibited a more porous MTA structure and more air gaps in the cavity wall, while V125E8 showed a relatively dense structure and fewer air gaps in the cavity wall. There was a significant difference in porosity (%) among the three groups (Figure 3(d)). DW showed the highest porosity, while V125E8 had the lowest.

### 3.4. Measurement of Stickiness, Flow Rate, and Contact Angle

**Figure 4:**
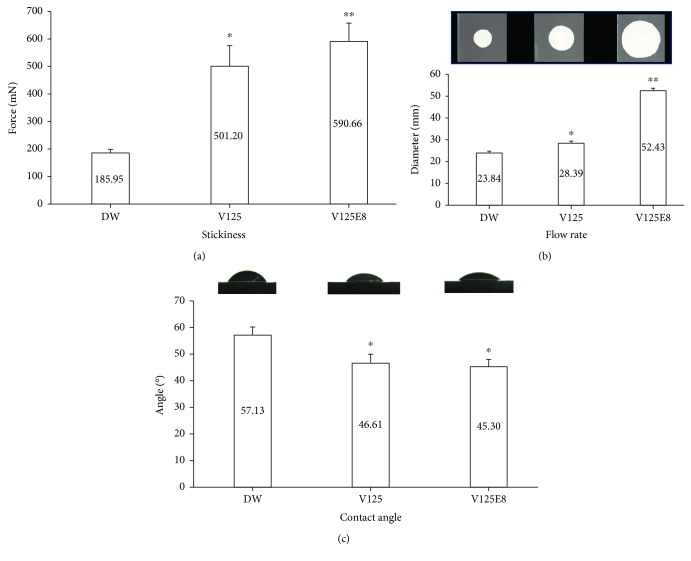
Evaluation of the flow rate, stickiness, and contact angle. (a, b) Flow rate and stickiness of the MTA mixture, respectively. (c) Contact angle of the DW, V125, and V125E8 solutions on the tooth disc. ^∗^*P* < 0.05 compared to the DW group; ^∗∗^*P* < 0.05 compared to both the DW and V125 groups.

The average maximum force on the probe tensile test is shown in Figure 4(a). The force reflects the capability of a mixture to stick two surfaces together. V125E8 had the stickiest properties among the groups, while DW demonstrated the lowest stickiness, with V125 in the middle (*P* < 0.05).

The flow rates of the three groups are presented in Figure 4(b). The flow rate of the MTA mixtures with the same L/P ratio exhibited significant differences according to liquid used (*P* < 0.05). The mean diameters of the pressed MTA mixtures between the two glasses were 23.84, 28.39, and 52.43 mm for the DW, V125, and V125E8 groups, respectively. V125E8 exhibited a significantly higher flow rate than V125 and DW.

The contact angle values of the ELP solutions (V125 and V125E8) were significantly lower than that of DW (*P* < 0.05) although there was no difference between the two ELP solutions (*P* > 0.05) (Figure 4(c)).

## 4. Discussion

We hypothesized that the V125E8 supplement would better adhere to dentin since octaglutamic acid was previously characterized as a hydroxyapatite binding motif [16, 21]. Our results affirm that this idea as supplementation of a specific ELP significantly enhanced the push-out bond strength of MTA to dentin and led to the improved performance of MTA in additional experiments related to bonding property. More MTA remnants were observed attached to the debonded dentin surface of adhesive failures in the V125E8 group than in the other groups (Figure 1). The V125E8 group also exhibited a closer and more rugged interface between the MTA mixtures and dentin, while DW showed a relatively larger gap and clearly separated interface under SEM observation (Figure 2). In addition, V125E8-supplemented MTA presented a smaller air space from the cavity wall and a more compact internal structure with fewer voids in the micro-CT evaluation (Figure 3).

We believe that increased bonding performance and a more compact structure of MTA will clinically result in great advantages. If MTA can acquire improved bonding properties to a tooth structure surrounding a defect, its retention to dentin and the hermetic sealing of the defect area will be enhanced. ELP-supplemented MTA with improved bonding properties may expand the application of MTA to large pulp exposures and root perforations by reducing the possibility of overextension. This may also be feasible through the improved physical properties and wash-out resistance of ELP-supplemented MTA shown in a previous study [15].

The reason for V125E8 supplementation increasing the bond strength of MTA to dentin can be deduced from the results of supporting experiments in this study. The increased flow rate and wettability of V125E8 may play an essential role in intimate contact with dentin (Figures 4(b) and 4(c)). Intimate contact by adequate adaptation is generally a prerequisite for ideal interactions with an adherend [22]. The high flow rate appears to come from the plasticizing action of V125E8 to promote particle dispersion [12, 23, 24]. Higher flowability may enable V125E8-supplemented MTA to infiltrate the dentinal tubules, which are visualized as tag-like structures at the interface between the MTA and dentin (Figure 2(i)). The high stickiness corresponding to the initial adhesion property may also serve to enhance the retention ability of MTA during the initial setting stage. We assume that the aforementioned flowability from the plasticizing effect of V125E8 and the surface charge of ELP, especially the sticky characteristics of glutamate in the case of V125E8, are related to the initial stickiness of the MTA mixture [11, 25]. In addition, the high calcium-binding property of carboxylic acid residues in V125E8 is believed to lead to good adhesion to dentin. The last possibility of increased bonding performance may come from denser structures of V125E8-supplemented MTA with fewer inside voids. The V125E8 group exhibited a significantly less porous structure in the micro-CT images compared to the other experimental groups, and the group also showed better adaptation to the cavity walls (Figures 3(a)–3(c)). We believe that this denser structure has a strong relationship with the higher strength and hardness of V125E8-supplemented MTA [12, 15]. Higher mechanical properties in the interface may cause a lower stress concentration effect and contribute to increased frictional resistance, thereby increasing the bond strength [26, 27].

In all experiments in this study, great care was taken to not apply any compaction pressure during the application of MTA in any cavity because such a force may alter the physical properties of MTA and contact with the cavity wall [25, 28]. This experimental setting could simulate clinical situations of pulp exposures and root perforations, where clinicians have to avoid pressure to the lesion to avoid extrusion of the MTA material in an undesired direction. Despite the consideration for clinical situations, a complete understanding of the true chemical reaction between ELP-supplemented MTA and dentin may be beyond the scope of this study. Further research is necessary to determine how ELP participates in the chemical reaction at the MTA-dentin interface.

## 5. Conclusion

Within the limitations of this study, V125E8 supplementation improves the bonding properties of MTA to the dentin surface. The sticky and highly flowable characteristics of V125E8-supplemented MTA may provide an intimate interface with dentin and supply a less porous cement structure. This study suggests that ELP-supplemented MTA could be used as an enhanced repair material for large discontinuities or defects that occur in the tooth structure, with improved bonding performance and physical properties.

## Data Availability

The data used to support the findings of this study are available from the corresponding author upon request.
